# Cost-effectiveness of Recurrent Dupuytren Contracture Treatment

**DOI:** 10.1001/jamanetworkopen.2020.19861

**Published:** 2020-10-08

**Authors:** Alfred P. Yoon, Robert L. Kane, David W. Hutton, Kevin C. Chung

**Affiliations:** 1 Section of Plastic Surgery, Department of Surgery, University of Michigan Medical School, Ann Arbor; 2 Health Management and Policy, University of Michigan School of Public Health, Ann Arbor

## Abstract

**Importance:**

Owing to its tendency to recur, Dupuytren contracture often requires multiple treatments, which places additional economic burden on health care. The likelihood of contracture recurrence varies not only with treatment but also with disease characteristics, such as contracture severity and location, but prior cost-effectiveness analyses of Dupuytren contracture treatments have not considered these patient-specific disease characteristics.

**Objective:**

To identify the most cost-effective treatment regimen for patients with recurrent Dupuytren contracture.

**Design, Setting, and Participants:**

This economic evaluation was conducted with state-transition microsimulation modeling using data from published studies and Medicare. A simulated cohort of 10 000 individuals with Dupuytren contracture was created. Patients could transition yearly between the following health states: symptom-free, symptomatic, and death. Available treatments were collagenase clostridium histolyticum injection, percutaneous needle aponeurotomy (PNA), and limited fasciectomy (LF); individuals randomly chose any treatment when symptomatic. Patients were limited to 3 rounds of treatment for a contracture affecting 1 joint, totaling 27 unique combinations. If the contracture recurred after 3 treatments, patients lived with the disease for the remainder of life.

**Exposures:**

PNA, collagenase clostridium histolyticum injection, or LF.

**Main Outcomes and Measures:**

Quality-adjusted life-years (QALYs), total costs (in US dollars), and incremental cost-effectiveness ratios (ICERs). A willingness-to-pay threshold of $100 000 per quality-adjusted life-year was used to assess cost-effectiveness.

**Results:**

For the base case scenario of a patient aged 60 years with recurrent, low-severity metacarpophalangeal (MCP) joint contracture, repeated PNA treatment was the only cost-effective treatment (2 PNA treatments followed by LF vs 3 PNA treatments, ICER [Monte Carlo SE]: $212 647/QALY [$36 000/QALY]). For recurrent high-severity MCP joint contractures, treatment regimens composed of PNA and LF were cost-effective (ICER [Monte Carlo SE], $93 932/QALY [$16 500/QALY]). LF was cost-effective for high-severity MCP joint contracture (ICER [Monte Carlo SE], $98 624/QALY [$26 233/QALY]). For recurrent proximal interphalangeal (PIP) joint contractures, PNA was the only cost-effective treatment, regardless of severity (eg, 2 PNA treatments followed by LF vs 3 PNA treatments for low-severity PIP joint contracture, ICER [Monte Carlo SE]: $263 726/QALY [$29 000/QALY]). Any combination with collagenase clostridium histolyticum injection compared with 3 PNA treatments had an ICER greater than $100 000 per QALY. Probabilistic sensitivity analysis estimated a 44%, 15%, 41%, and 52% chance of a regimen consisting of only PNA being cost-effective in low-severity MCP, high-severity MCP, low-severity PIP, and high-severity PIP joint contractures, respectively.

**Conclusions and Relevance:**

The results of this study suggest that LF is a cost-effective intervention for recurrent high-severity MCP joint contractures. For recurrent low-severity MCP joint contractures and PIP joint contractures of all severity levels, PNA was the only cost-effective intervention. Collagenase clostridium histolyticum injections were not a cost-effective intervention for recurrent Dupuytren contracture and should not be preferred over PNA or LF.

## Key Points

**Question:**

What is the most cost-effective treatment regimen for recurrent Dupuytren contracture based on disease characteristics?

**Findings:**

In this economic evaluation, limited fasciectomy was a cost-effective treatment for recurrent severe (ie, >45°) Dupuytren contracture at the metacarpophalangeal joint compared with percutaneous needle aponeurotomy. In low-severity metacarpophalangeal joint and proximal interphalangeal joint contractures, percutaneous needle aponeurotomy was the only cost-effective intervention.

**Meaning:**

In this study, limited fasciectomy was cost-effective for treating recurrent, severe metacarpophalangeal joint contractures, but percutaneous needle aponeurotomy was the only cost-effective treatment for recurrent low-severity metacarpophalangeal joint contractures and recurrent proximal interphalangeal joint contractures.

## Teaser

This economic evaluation identifies the most cost-effective treatment regimen for patients with recurrent Dupuytren contracture.

## Introduction

Dupuytren contracture is a late manifestation of Dupuytren disease, a chronic fibroproliferative disorder of the palmar fascia that preferentially affects the ring and small fingers.^1,2^ Its prevalence is between 1% and 32% in North American and European populations.^3,4^ In England, the yearly national estimated treatment cost for Dupuytren contracture is £41 576 141 ($52 047 092).^5^ Dupuytren contracture is commonly managed with limited fasciectomy (LF), a surgical procedure that removes diseased connective tissue.^6^ However, less invasive alternatives, such as enzymatic release with collagenase clostridium histolyticum (CCH) injection and manual division of cords using percutaneous needle aponeurotomy (PNA), have both demonstrated efficacy.^7^ CCH and PNA are associated with faster recovery time and consume fewer resources than LF.^8,9,10^ However, the likelihood that the contracture will recur following treatment may be greater for PNA and CCH.^7,11^ In addition, some studies show that PNA and CCH are less effective for treating proximal interphalangeal (PIP) joint contractures compared with LF^11,12^ and that severe contractures are more amenable to treatment with LF.^10,13,14^ Balancing these trade-offs can make treatment selection challenging, especially when no formal treatment guidelines exist. Consequently, both nonsurgical and surgical treatments are applied in various combinations to the same joint when Dupuytren contracture recurs.^15,16^ Furthermore, as treatment costs for LF, PNA, and CCH differ substantially,^8^ identifying the most cost-effective treatment regimen has the potential to considerably reduce health care expenditure.

Because recurrence and treatment success vary with contracture severity and affected joint type,^17,18,19,20^ it is likely that these disease characteristics are associated with the cost-effectiveness of Dupuytren contracture treatments. Prior cost-effectiveness studies on Dupuytren contracture treatment generated conflicting conclusions.^8,13,21,22^ The National Health Service in the United Kingdom suggested that LF is the most cost-effective intervention,^13^ yet other studies found the opposite.^8,21^ To our knowledge, no study has incorporated both contracture severity and affected joint type when modeling the cost-effectiveness of treating recurrent Dupuytren contracture. Furthermore, previous studies used cohort models, such as decision trees and Markov models, that limited their ability to incorporate patient-level parameters. A microsimulation model presents an opportunity to integrate these patient-level characteristics to more accurately project treatment costs and health effects gained for different Dupuytren contracture phenotypes.^23^ Unlike prior studies, a microsimulation can model individualized disease outcomes and probabilities that depend on cumulative events in a patient’s history, such as contracture recurrence.

The economic burden associated with treating Dupuytren contracture increased by almost 50% between 1998 and 2011 and is projected to increase as the population ages.^22^ The aim of this study was to identify cost-effective treatment regimens for recurrent Dupuytren contracture based on patient-level characteristics that have known associations with treatment outcome and recurrence. To accomplish this, we conducted a microsimulation economic analysis incorporating patients’ contracture severity, affected joint type, and number of joints affected.

## Methods

The methods and presentation of study findings adhered to the Consolidated Health Economic Evaluation Reporting Standards (CHEERS) reporting guideline. According to the University of Michigan institutional review board, this study fell under the University of Michigan’s policy for research using publicly available data sets. Under this policy and in accordance with federal regulations for human subjects research (45 CFR Part 46), institutional review board approval was not required because the data cannot be tracked to a human participant.

### Model Design

We compared the cost-effectiveness of 3 interventions (CCH, PNA, and LF) using state-transition microsimulation modeling with a lifetime horizon. The model incorporated both societal and health-sector perspectives in the context of health care in the United States. The base case scenario was a patient aged 60 years with low-severity MCP joint contracture. Contractures of less than 45° from full finger extension were defined as low severity and those greater than 45° as high severity. Scenarios considered in the model were high-severity MCP joint, low-severity PIP joint, and high-severity PIP joint contractures.

**Figure 1.**
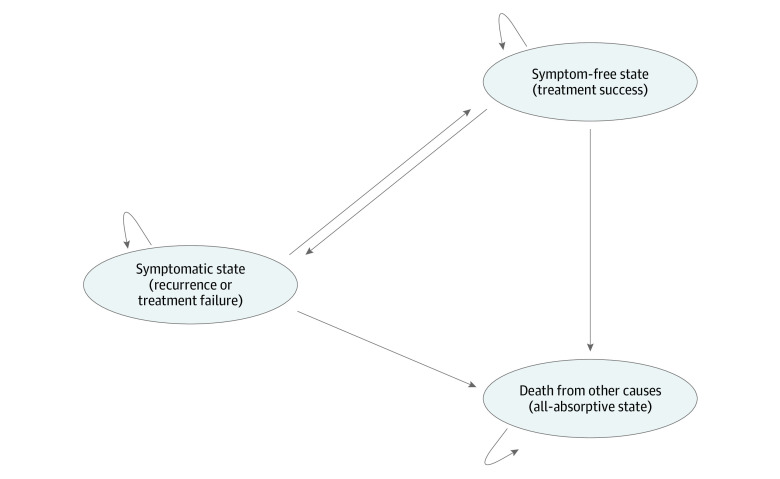
Markov Model Structure for State-Transition Microsimulation Hooked arrows indicate that patients can remain in the same state each year.

The overall model structure included the 3 following health states: (1) symptom-free state (ie, treatment success), (2) symptomatic state (ie, recurrence or treatment failure) and (3) death from other causes (Figure 1). Every patient entered the model with diagnosed Dupuytren contracture desiring intervention. The cycle length for the microsimulation was 1 year with a lifetime horizon, indicating that all simulated patients could transition between health states once per year until death. Likewise, this cycle length permits patients in the symptomatic state to receive 1 treatment per year. At the start of the simulation, patients underwent CCH, PNA, or LF as their first treatment while in the symptomatic state. Then, depending on the probability of a treatment’s success relative to patient characteristics, the patient transitioned to the symptom-free state if the treatment succeeded or remained in the symptomatic state if the treatment failed. Patients who achieved a symptom-free state could revert back to the symptomatic state each year, depending on the probability of recurrence, which represented the risk of developing recurrent contracture. The probability for a contracture to recur after treatment depended on the chosen treatment and contracture characteristics, similar to the probability of treatment success. Patients could attempt a maximum of 3 treatments for a single contracture, but if the contracture recurred after the third treatment, the patient lived with the disease until death, accruing reduced utilities each year. With a maximum of 3 treatments chosen at random, 27 unique treatment regimens comprised of CCH, PNA, and LF were possible.

Contingent on age-dependent mortality rates,^24^ the patient could transition to an all-absorptive death state (ie, the patient cannot come out of the death state) where no cost or utility accrued. For each joint type and severity, 10 000 unique simulated patients living with recurrent Dupuytren contracture were generated. Cost and health outcomes were discounted by 3% each year. Patients accrued cost and decreased utilities associated with treatments and for time spent in the symptomatic state.

### Transition Probabilities

Based on published data, we calculated transition probabilities for treatment success and recurrence for each intervention and associated joint type and contracture severity. The studies included for the calculation of transition parameters were published between 2002 and 2018, with a mean follow-up time of 1.6 years (range: 1 month to 2 years).^9,10,11,14,17,20,25,26,27,28,29,30,31,32,33,34,35,36,37,38,39^ In accordance with previous randomized clinical trials, we included studies that defined treatment success as less than 5° of residual contracture at 30-day follow-up. Recurrence was defined as return of a contracture greater than 20°.^17,26,28,40^ First, a baseline weighted mean probability of success and recurrence for each intervention was calculated. Then, these probabilities were adjusted by the relative risk of success and recurrence contingent on the affected joint type and contracture severity (Table 1).^2,4,8,9,11,12,13,14,17,20,21,24,25,28,29,30,32,33,34,35,36,37,38,39,40,41,42,43,44,45,46,47,48,49^

**Table 1.** List of Model Variables for Base Case Scenario

Variable	Base case value	Low^a^		High^a^		Distribution	Source
**Demographic characteristics**							
Age, y	60	45		7		Uniform	Van Rijssen et al,^11^ 2012; Brazzelli et al,^13^ 2015
Mortality per year, %	0.87	0.18		12.9		Uniform	US Centers for Disease Control and Prevention^24^
**Transition probabilities**							
CCH success							
MCP joint							
Low severity	0.61	0.52		0.71		β	Hurst et al,^28^ 2009; Badalamente et al,^41^ 2015; Bainbridge et al,^42^ 2012
High severity	0.39	0.33		0.45		β	Muppavarapu et al.^14^ 2015; Hurst et al,^28^ 2009; Witthaut et al,^30^ 2013; Badalamente et al,^41^ 2015; Bainbridge et al,^42^ 2012
PIP joint							
Low severity	0.33	0.28		0.38		β	Naam et al,^9^ 2013; Zhou et al,^12^ 2015; Badalamente et al,^17^ 2002; Peimer et al,^20^ 2015; Hurst et al,^28^ 2009; Stromberg et al,^29^ 2018; Witthaut et al,^30^ 2013; Nayar et al,^35^ 2019; Gaston et al,^36^ 2015; Hansen et al,^37^ 2017; Gilpin et al,^38^ 2010; Mickelson et al,^40^ 2014; Badalamente et al,^41^ 2015; Bainbridge et al,^42^ 2012; Coleman et al,^43^ 2014; Warwick et al,^44^ 2016
High severity	0.13	0.11		0.15		β	Naam et al,^9^ 2013; Zhou et al,^12^ 2015; Muppavarapu et al,^14^ 2015; Badalamente et al,^17^ 2002; Peimer et al,^20^ 2015; Hurst et al,^28^ 2009; Stromberg et al,^29^ 2018; Witthaut et al,^30^ 2013; Nayar et al,^35^ 2019; Gaston et al,^36^ 2015; Hansen et al,^37^ 2017; Gilpin et al,^38^ 2010; Mickelson et al,^40^ 2014; Badalamente et al,^41^ 2015; Bainbridge et al,^42^ 2012; Coleman et al,^43^ 2014; Warwick et al,^44^ 2016
**CCH recurrence**							
MCP joint							
Low severity	0.25	0.21		0.29		β	Muppavarapu et al,^14^ 2015; Badalamente et al,^17^ 2002; Peimer et al,^20^ 2015; Stromberg et al,^29^ 2018; Nayar et al,^34^ 2019; Hansen et al,^37^ 2017; McMahon et al,^39^ 2013
High severity	0.25	0.21		0.29		β	Muppavarapu et al,^14^ 2015; Badalamente et al,^17^ 2002; Peimer et al,^20^ 2015; Stromberg et al,^29^ 2018; Nayar et al,^34^ 2019; Hansen et al,^37^ 2017; McMahon et al,^39^ 2013
PIP joint							
Low severity	0.48	0.41		0.55		β	Muppavarapu et al,^14^ 2015; Badalamente et al,^17^ 2002; Peimer et al,^20^ 2015; Stromberg et al,^29^ 2018; Nayar et al,^34^ 2019; Hansen et al,^37^ 2017; McMahon et al,^39^ 2013
High severity	0.48	0.41		0.55		β	Muppavarapu et al,^14^ 2015; Badalamente et al,^17^ 2002; Peimer et al,^20^ 2015; Stromberg et al,^29^ 2018; Nayar et al,^34^ 2019; Hansen et al,^37^ 2017; McMahon et al,^39^ 2013
**PNA success**							
MCP joint							
Low severity	0.58	0.49		0.67		β	Stromberg et al,^29^ 2018; Abe et al,^32^ 2015
High severity	0.33	0.28		0.38		β	Stromberg et al,^29^ 2018; Abe et al,^32^ 2015
PIP joint							
Low severity	0.37	0.31		0.43		β	Stromberg et al,^29^ 2018; Abe et al,^32^ 2015
High severity	0.21	0.18		0.24		β	Stromberg et al,^29^ 2018; Abe et al,^32^ 2015
**PNA recurrence**							
MCP joint							
Low severity	0.26	0.22		0.30		β	Stromberg et al,^29^ 2018; Abe et al,^32^ 2015; Selles et al,^33^ 2018
High severity	0.26	0.22		0.30		β	Stromberg et al,^29^ 2018; Abe et al,^32^ 2015; Selles et al,^33^ 2018
PIP joint							
Low severity	0.40	0.34		0.46		β	Stromberg et al,^29^ 2018; Abe et al,^32^ 2015; Selles et al,^33^ 2018
High severity	0.40	0.34		0.46		β	Stromberg et al,^29^ 2018; Abe et al,^32^ 2015; Selles et al,^33^ 2018
**LF success**							
MCP joint							
Low severity	0.71	0.60		0.82		β	Zhou et al,^12^ 2015; Muppavarapu et al,^14^ 2015
High severity	0.61	0.52		0.70		β	Zhou et al,^12^ 2015; Muppavarapu et al,^14^ 2015
PIP joint							
Low severity	0.59	0.50		0.68		β	Zhou et al,^12^ 2015; Muppavarapu et al,^14^ 2015
High severity	0.25	0.21		0.29		β	Zhou et al,^12^ 2015; Muppavarapu et al,1^4^ 2015
**LF recurrence**							
MCP joint							
Low severity	0.18	0.15		0.21		Beta	Selles et al,^33^ 2018
High severity	0.18	0.15		0.21		Beta	Selles et al,^33^ 2018
PIP joint							
Low severity	0.24	0.20		0.28		Beta	Selles et al,^33^ 2018
High severity	0.24	0.20		0.28		Beta	Selles et al,^33^ 2018
**Direct costs, $**							
CCH							
Clinic visit	172.26	146.42		198.10		Normal	CMS,^45^ *CPT* code 20527
Medication	5400.00	4590.00		6210.00		Normal	Chen et al,^8^ 2011
Manipulation visit	209.02	177.67		240.37		Normal	CMS,^45^ *CPT* code 26341
Hand therapy	314.62	221.64		407.60		Normal	Expert opinion, CMS,^45^ splint (*CPT* code 97760), 2 visits (*CPT* code 97165), adjusting splint (*CPT* code 97763), and manual therapy (*CPT* code 97140)
PNA							
Procedure	322.91	274.47		371.35		Normal	CMS,^45^ *CPT* code 26040
Hand therapy	673.57	487.61		859.53		Normal	Expert opinion; CMS, ^45^ splint (*CPT* code 97760), 5 visits (*CPT* code 97165), 2 visits to adjust splint (*CPT* code 97763), 2 visits for manual therapy (*CPT* code 97140)
LF							
Procedure	1149.29	976.90		1321.68		Normal	CMS,^45^ *CPT* code 26123
Anesthesia	245.00	208.25		281.75		Normal	Chen et al,^8^ 2011; CMS^45^
Facility fee	2623.34	2229.84		3016.84		Normal	CMS^45^
Hand therapy	1394.35	1115.41		1859.25		Normal	Expert opinion; CMS^45^
Complication cost, $							
Tendon injury	3479.56	2957.63		4001.49		Normal	CMS,^45^ *CPT* code 26350
Admission	7987.00	6788.95		9185.05		Normal	CMS,^45^ DRG 514
Medication allergy	176.23	149.80		202.66		Normal	CMS,^45^ *CPT* code 99285
Skin tear	75.32	64.02		86.62		Normal	CMS,^45^ *CPT* code 99213 for 3 visits
Infection	2746.26	2334.32		3158.20		Normal	CMS, ^45^ *CPT* code 10180
CRPS	2000.33	1700.28		2300.38		Normal	Baltzer et al,^21^ 2013; CMS^45^
Nerve injury	5435.18	4619.90		6250.46		Normal	CMS^45^
Artery injury	3581.64	3044.39		4118.89		Normal	CMS^45^
Hematoma	1658.78	1409.96		1907.60		Normal	CMS^45^
Complication rate, %							
CCH							
Tendon injury	0.38	0.34		0.46		β	Gaston et al,^36^ 2015; McMahon et al,^39^ 2013; Badalamente et al,^41^ 2015; Coleman et al,^43^ 2014
Admission	1.56	1.33		1.79		β	Coleman et al,^43^ 2014
Adverse reaction	0.26	0.22		0.30		β	Badalamente et al,^17^ 2002; Gaston et al,^36^ 2015
Skin tear	3.97	3.37		4.57		β	Alberton et al,^25^ 2014; Warwick et al,^44^ 2016
PNA							
Tendon injury	0.38	0.32		0.44		β	Bainbridge et al.^46^ 2012; Herrera et al,^47^ 2015
Infection	1.55	1.32		1.78		β	Herrera et al,^47^ 2015
CRPS	0.52	0.44		0.60		β	Herrera et al,^47^ 2015
Nerve injury	0.38	0.32		0.44		β	Bainbridge et al,^42^ 2012; Herrera et al,^47^ 2015
Artery injury	0.91	0.77		1.05		β	Bainbridge et al,^42^ 2012
LF							
Tendon injury	0.17	0.14		0.20		β	Bainbridge et al,^42^ 2012
Admission	1.13	0.96		1.30		β	Bainbridge et al,^42^ 2012
Infection	1.28	1.09		1.47		β	Van Rijssen et al,^11^ 2012
Nerve injury	2.16	1.84		2.48		β	Van Rijssen et al,^11^ 2012; Zhou et al,^2^ 2015; Bainbridge et al,^46^ 2012
Artery injury	0.95	0.81		1.09		β	Bainbridge et al,^46^ 2012
Hematoma	1.28	1.09		1.47		β	Van Rijssen et al,^11^ 2012
**Indirect costs**^**b**^							
Time off work, d							
CCH	1	0		3		Normal	Naam et al,^9^ 2013; expert opinion
PNA	1	0		14		Normal	Naam et al,^9^ 2013; expert opinion
LF	37	14		60		Normal	Naam et al,^9^ 2013; expert opinion
**Utility while in symptomatic state**							
MCP							
Low severity	0.969	0.824		1.000		β	Gu et al,^48^ 2013
High severity	0.938	0.891		0.985		β	Gu et al,^48^ 2013
PIP							
Low severity	0.970	0.922		1.000		β	Gu et al,^48^ 2013
High severity	0.942	0.895		0.989		β	Gu et al,^48^ 2013

Abbreviations: CCH, collagenase clostridium histolyticum; CMS, Centers for Medicare & Medicaid Services; *CPT*, *Current Procedural Terminology*; CRPS, complex regional pain syndrome; DRG, diagnosis related group; LF, limited fasciectomy; MCP, metacarpophalangeal; PIP, proximal interphalangeal; PNA, percutaneous needle aponeurotomy.

^a^ These values used for sensitivity analysis. The distributions for the probabilistic sensitivity analysis are parameterized as follows: all distributions are set so as to have approximately 95% of the distribution lie between the low and high values, with β distributions based on 100 data points informing the estimate and using a noninformative prior and all normal distributions truncated to be greater than 0 (and <1 for reduction in wages and utilities). All distributions are assumed to be independent.

^b^ Based on the 2018 median income of $63 179, according to the Bureau of Labor Statistics.^49^

### Cost and Health Outcomes

Procedure cost, anesthesia cost, facility fee, hand therapy, collagenase medication, additional cost from complications, and collagenase injection and manipulation clinic visits were considered direct costs. Physician fees were derived from the 2019 National Physician Fee schedule using *Current Procedural Terminology* (*CPT*) codes (Table 1).^45^ Facility fees were determined from the Medicare Outpatient Prospective Payer System, and collagenase costs were gleaned from literature review.^8^ Indirect costs, such as wages lost from time off work after each intervention, were based on 2018 US median income.^49^ Only complications associated with substantial cost or long-term impairment in quality of life were included. Rates of complication were derived from the literature.^10,12,17,20,25,36,39,41,42,43,44,46,47^ The frequency and type of hand therapy associated with each treatment were provided by hand therapists at Michigan Medicine, and costs were calculated using Medicare fee schedules.

Health utilities were calculated using a previously described discrete choice experiment (Table 1).^48^ A weighted mean utility based on Dupuytren disease prevalence of each finger was calculated for the base case single joint analysis.^27^ For the 2-finger analysis, utilities were calculated for ring and small finger Dupuytren contractures.

### Model Assumptions and Validation

The model assumed that the probability of immediate treatment failure was the inverse of the success rate for each intervention. Utilities lost from complications were not considered because most complications were relatively rare and short-term. In addition, the discrete choice experiment formula used does not consider complications when calculating utilities. In alignment with general practice, we assumed that PNA and CCH were performed in clinic, whereas LF was performed in the operating room under general anesthesia. Because of the 1-year cycle length, patients who recurred or had treatment failure accumulated a discounted utility for an entire year. The symptomatic state utility was identical regardless of treatment. Furthermore, because of limited outcome data for treating recurrent contractures, we assumed that the probability of success and recurrence for each treatment type would remain constant whether used as the initial treatment vs treatment for recurrence. Lastly, the model assumed that patients sought treatment for 3 episodes of recurrent Dupuytren contracture, although in reality some patients may defer treatment much earlier.

Several hand surgeons with expertise in Dupuytren contracture confirmed the face validity of the model by evaluating the model structure, data sources, assumptions, and results. The accuracy of the code was validated by code breaks, independent line-by-line code review by 2 of us (A.P.Y. and D.W.H.), and sensitivity analyses. External validity was evaluated by comparing the model’s estimation of mean recurrence time to reported literature times.

### Statistical Analysis

The primary outcome measure was the incremental cost-effectiveness ratio (ICER) among the 27 unique treatment regimens. The ICER was calculated by dividing the difference in total cost between 2 treatment regimens by the corresponding difference in quality-adjusted life-years (QALY) gained. The net monetary benefit (NMB) for each treatment regimen was estimated using the formula^50^ NMB = λ × Δ_e_ – Δ_c_, in which *λ* is the willingness-to-pay threshold, *e* is effectiveness, and *c* is cost. One-way sensitivity analysis varying all model parameters was conducted to identify the most influential factors in determining cost-effectiveness (Table 1). A willingness-to-pay threshold of $100 000 per QALY was used as the cost-effectiveness threshold.^51,52^ Probabilistic sensitivity analysis simulating uncertainty in all parameters simultaneously was conducted to generate cost-effectiveness acceptability curves using a Monte Carlo simulation modeling 10 000 patients with randomly selected model parameters within a clinically feasible distribution. Uncertain intervals were represented by standard deviation or Monte Carlo Standard Error (MCSE). The modeling and analysis were conducted with R Studio version 1.2.5033 (R Project for Statistical Computing) adapting a previously published microsimulation script.^53^ Statistical significance was set at a 2-tailed *P* < .05.

## Results

In a simulated cohort of 10 000 patients with high-severity MCP joint contractures, the mean (SD) time to first recurrence was 26 (28.9) months after PNA and 29 (31.7) months after CCH. These were comparable to published recurrence times of 19 to 33 months for PNA and 24 months for CCH.^10,54^ PIP joint contractures had shorter mean (SD) time to first recurrence than MCP joint contractures (PNA: 5.0 [2.5] years vs 7.5 [4.4] years; *P* < .001; LF: 7.4 [4.7] years vs 10.7 [5.8] years; *P* < .001).

PNA for all 3 treatments was the least expensive strategy (eg, mean [SD] total cost for low-severity MCP joint contracture: $3339 [$544]), whereas LF for all 3 treatments was the most expensive (eg, mean [SD] total cost for low-severity MCP joint contracture: $25 419 [$6785]). Mean (SD) lifetime accumulated QALYs were slightly higher for having all 3 treatments be LF compared with PNA (eg, low-severity MCP joint contracture: 15.17 [4.98] vs 15.08 [4.95]), whereas, for every Dupuytren contracture phenotype, the cost of all 3 treatments being LF was more than 8-fold higher than PNA (Table 2; eFigure 1 in the Supplement). Having PNA for all 3 treatments was the only cost-effective regimen for every joint type and severity level, except for high-severity MCP joint contracture, assuming a decision-maker is not willing to pay more than $200 000 per QALY gained (Table 2). In high-severity MCP joint contractures, starting with PNA the first 2 times and moving to LF after the second recurrence had an ICER of $93 932 (MCSE, $16 500) per QALY. Having LF for all 3 treatments among patients with high-severity MCP joint contracture had an ICER of $98 624 (MCSE, $26 333) per QALY (Table 2).

**Table 2.** QALY and ICER for Single-Joint Contracture From Societal Perspective

Joint type and severity	Treatment combination^a^	Mean (SD)		ICER (MCSE), $/QALY^b^
		Total cost, $	Lifetime QALYs	
MCP joint				
Low severity	PNA-PNA-PNA	3339 (544)	15.09 (4.95)	Reference case
	PNA-PNA-LF	9448 (4003)	15.12 (4.97)	212 647 (36 000)
	LF-LF-LF	25 419 (6785)	15.17 (4.98)	293 592 (48 000)
High severity	PNA-PNA-PNA	3512 (462)	14.64 (4.80)	Reference case
	PNA-PNA-LF	11 225 (3653)	14.72 (4.83)	93 932 (16 500)
	LF-LF-LF	26 527 (6879)	14.88 (4.87)	98 624 (26 333)
PIP joint				
Low severity	PNA-PNA-PNA	3567 (399)	15.03 (4.95)	Reference case
	PNA-PNA-LF	11 789 (3217)	15.06 (4.95)	263 726 (29 000)
	LF-LF-LF	27 940 (6465)	15.12 (4.97)	263 427 (64 000)
High severity	PNA-PNA-PNA	3640 (338)	14.59 (4.80)	Reference case
	PNA-PNA-LF	12 574 (2649)	14.62 (4.81)	421 843 (24 000)
	LF-LF-LF	31 472 (5307)	14.66 (4.82)	408 244 (25 500)

Abbreviations: CCH, collagenase clostridium histolyticum; ICER, incremental cost-effectiveness ratio; LF, limited fasciectomy; MCP, metacarpophalangeal; MCSE, Monte Carlo standard error; PNA, percutaneous needle aponeurotomy; QALY, quality-adjusted life-year.

^a^ A total of 27 treatment sequences were modeled, as follows: (1) CCH-PNA-LF, (2) CCH-LF-PNA, (3) PNA-CCH-LF, (4) PNA-LF-CCH, (5) LF-CCH- PNA, (6) LF-PNA-CCH, (7) CCH-CCH-PNA, (8) CCH-CCH-LF, (9) CCH-PNA-CCH, (10) CCH-LF-CCH, (11) PNA-PNA-LF, (12) PNA-PNA-CCH, (13) PNA-CCH-PNA, (14) PNA-LF-PNA, (15) LF-LF-CCH, (16) LF-LF-PNA, (17) LF-CCH-LF, (18) LF-PNA-LF, (19) PNA-CCH-CCH, (20) LF-CCH-CCH, (21) CCH-PNA-PNA, (22) LF-PNA-PNA, (23) CCH-LF-LF, (24) PNA-LF-LF, (25) CCH-CCH-CCH, (26) PNA-PNA-PNA, and (27) LF-LF-LF.

^b^ ICER compared with next best treatment combination with highest net monetary benefit.

**Figure 2.**
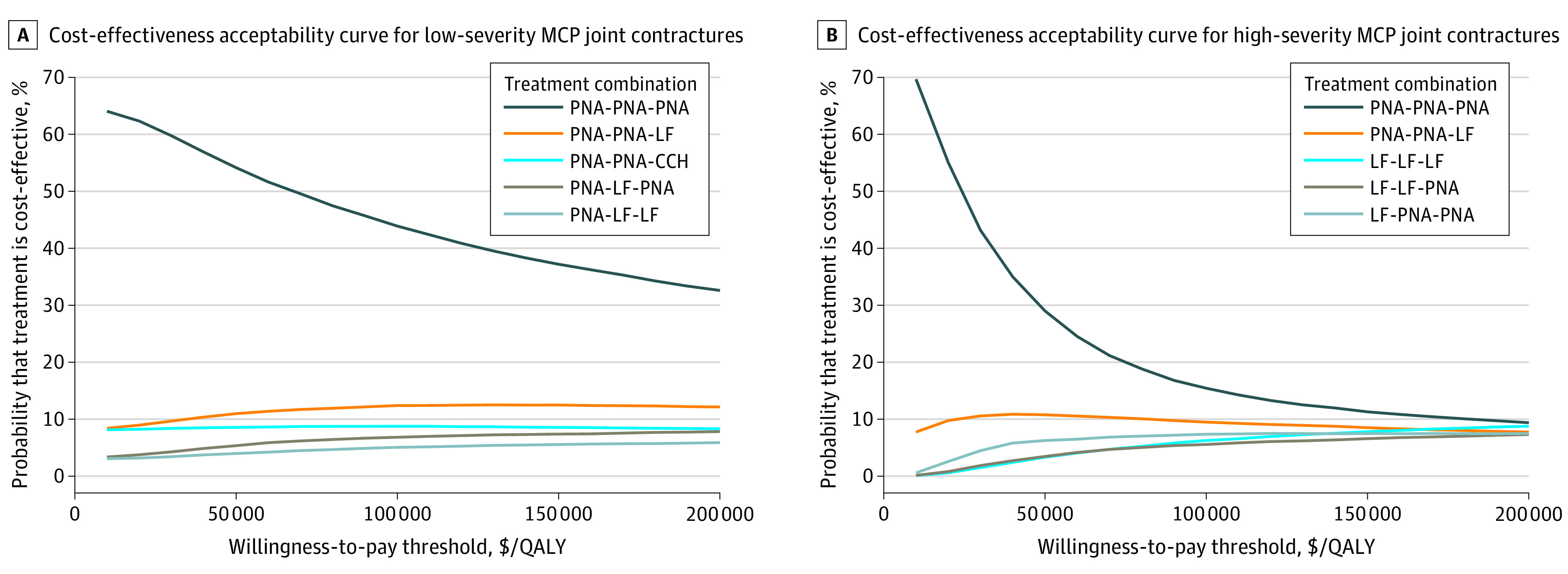
Cost-effectiveness Acceptability Curve for Metacarpophalangeal (MCP) Joint Contractures At a willingness-to-pay threshold of $100 000 per quality-adjusted life-year (QALY), 3 percutaneous needle aponeurotomy (PNA) treatments (PNA-PNA-PNA) had a 44% and 15% chance of being the most cost-effective treatment combination compared with the remaining 26 treatment sequences in low-severity and high-severity MCP joint contractures, respectively. CCH indicates collagenase clostridium histolyticum injection; and LF, limited fasciectomy.

**Figure 3.**
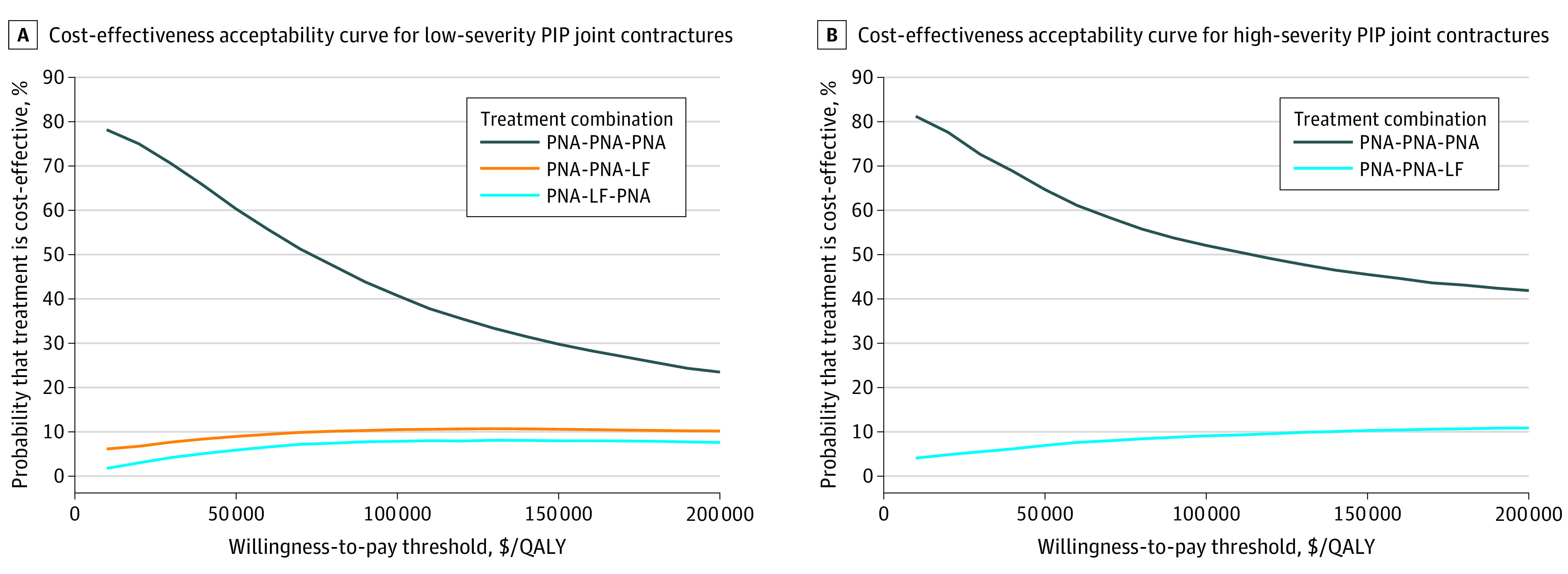
Cost-effectiveness Acceptability Curve for Proximal Interphalangeal (PIP) Joint Contractures At a willingness-to-pay threshold of $100 000 per quality-adjusted life-year (QALY), 3 percutaneous needle aponeurotomy (PNA) treatments (PNA-PNA-PNA) had a 41% and 52% chance of being the most cost-effective treatment combination compared with the remaining 26 treatment sequences in low-severity and high-severity MCP joint contractures, respectively. LF indicates limited fasciectomy.

One-way sensitivity analysis revealed that the utility during symptomatic state was the most important parameter influencing cost-effectiveness in every joint type and severity, except high-severity PIP joint contractures (eTable 1 in the Supplement). When the symptomatic state utility was 5% lower than the base case utility, treatment combinations of PNA and LF became cost-effective for low-severity MCP and PIP joint contractures. PNA for all 3 episodes remained the most cost-effective treatment combination for high-severity PIP joint contractures irrespective of variations in any single parameter. LF was favored for high-severity MCP joint contracture with the following characteristics: younger patients, lower wage losses, lower facility fee, lower PNA success rate, and higher LF success rate. The contrary of these characteristics favored PNA (eTable 1 in the Supplement). Probabilistic sensitivity analysis revealed that, at a willingness-to-pay threshold of $100 000 per QALY, PNA for all 3 treatments had a 44%, 15%, 41%, and 52% chance of being the most cost-effective intervention in low-severity MCP joint contracture, high-severity MCP joint contracture, low-severity PIP joint contracture, and high-severity PIP joint contracture, respectively (Figure 2 and Figure 3). As the willingness-to-pay threshold increased, it became less likely that PNA for all 3 treatments would be the most cost-effective intervention, although a better alternative strategy was unclear. A separate analysis was conducted to simulate cost-effectiveness when PNA was performed in the operating room (data not shown). Despite the added cost, having PNA for all 3 treatments continued to be the only cost-effective treatment regimen for low-severity MCP, low-severity PIP, and high-severity PIP joint contractures. In high-severity MCP joint contractures, any treatment regimen that involved LF at least once had an ICER less than $100 000 per QALY.

From a health-sector perspective, similar to societal perspective results, treatment regimens with PNA and LF were cost-effective for high-severity MCP joint contractures, whereas repeated PNA was the only cost-effective strategy for the remaining joint and severity types (eTable 2 in the Supplement). When the model simulated 2 fingers (ring and small fingers) affected by Dupuytren contracture simultaneously, any treatment combination of LF and PNA remained cost-effective in high-severity MCP joint contractures (eTable 3 in the Supplement). Two rounds of PNA followed by LF had lower ICERs for patients with 2-finger involvement vs 1-finger involvement (ICER [MCSE] for low-severity MCP: $120 999/QALY [$36 000/QALY] vs $212 647/QALY [$36 000/QALY]; low-severity PIP: $127 131/QALY [$14 500/QALY] vs $263 726/QALY [$29 000/QALY]) (Table 2; eTable 3 in the Supplement). Probabilistic sensitivity analysis revealed a 34%, 10%, 20%, and 40% chance of having all 3 treatments be PNA being the most cost-effective treatment combination for patients with 2-finger low-severity MCP, high-severity MCP, low-severity PIP, and high-severity PIP contractures, respectively (eFigure 2 and eFigure 3 in the Supplement).

## Discussion

This study suggests that the most cost-effective treatment regimen for recurrent Dupuytren contracture is associated with joint type, contracture severity, and number of fingers affected. In single-digit high-severity MCP joint contractures, PNA for the initial treatment followed by LF to manage recurrence was a cost-effective treatment regimen. In the remaining joint and severity types, PNA was the only cost-effective treatment for recurrent contractures. Similar overall findings were observed in 2-finger disease; however, if the patient's quality of life is severely affected by Dupuytren contracture, LF may become a cost-effective treatment even for low-severity MCP and PIP joint contractures. Treatment regimens involving CCH were not found to be cost-effective in any scenario.

Few cost-effectiveness analyses have rigorously analyzed Dupuytren contracture treatments. A 2011 study from the United States^8^ concluded that LF and CCH are not cost-effective compared with PNA using a decision-tree model. However, this study did not account for recurrence, lacked probabilistic sensitivity analysis, and may have overinflated utilities. Nevertheless, our results support this study’s findings that the current price of CCH in the United States does not make it a cost-effective agent. Similarly, our microsimulation also found that PNA was the most cost-effective method in low-severity MCP joint contractures. On the contrary, a UK study concluded that LF is the most cost-effective treatment because of greater QALY gain and lower recurrence rate compared with PNA or CCH.^13^ However, this should be interpreted with caution given that the cost of LF in the UK ($2700) is considerably cheaper than in the US ($5411).^13^ Furthermore, this study did not consider affected joint type and limited the third-line treatment to LF. Nonetheless, the similarity of their findings to our own adds face validity to our microsimulation, demonstrating that despite the higher cost of LF, patients accrued the most QALYs compared with PNA and CCH.

Our review of the literature found that the risk-adjusted probabilities of treatment success for high-severity PIP joint contractures were approximately 13% for CCH, 21% for PNA, and 25% for LF. Overall, treatment success was lower for PIP joints than MCP joints in every treatment. Because PNA is the least expensive intervention and no treatment is particularly effective for PIP joints, this may explain why repeated PNA was the only cost-effective treatment regimen for single PIP joint Dupuytren contracture in our model. Estimated utilities after non–life-threatening conditions such as Dupuytren contracture are generally high, even when disease-specific quantitative techniques, such as discrete choice experiments, are implemented.^48^ When multiple digits are involved, the health utility correspondingly decreases. However, the treatment costs for LF increase by a relatively nominal amount owing to longer operative time and slightly higher professional fees. Consequently, for patients with 2-finger Dupuytren contracture whose quality of life is severely impacted, LF may be cost-effective, as seen in our 2-finger disease 1-way sensitivity analysis. Furthermore, we believe this interpretation can be cautiously extrapolated to patients with multiple fingers (ie, >2) affected, making LF a cost-effective treatment for those patients.

### Limitations

The findings of this study should be interpreted within the context of some limitations. Because no randomized clinical trial with a direct comparison of all 3 treatments exists, the transition probabilities were derived from studies that compared 2 treatments or a single treatment to a placebo. Both prospective and retrospective studies were included to gather sufficient data to calculate transition probabilities based on both joint type and contracture severity. This can introduce heterogeneity into the model because the treatment arms of each study will likely have patients with different characteristics. To address these uncertainties and heterogeneity, we conducted both deterministic and probabilistic sensitivity analyses, varying the model parameters over feasible ranges. In addition, there may be other patient-level risk factors that affect treatment success and recurrence, such as Garrod pads, bilateral involvement, age of onset, and family history.^55^ These factors were ultimately excluded from the model owing to insufficient high-quality evidence. Assessing recurrence of Dupuytren contracture can be challenging because of similarities in presentation with scar contractures, especially in recurrences after LF. To maximize the inclusion of only true Dupuytren contracture recurrence rates in the literature, we adopted strict inclusion criteria for recurrences as contractures greater than 20°.

Our findings should not be generalized to countries outside of the United States because of different health care costs and mortality rates. This study benefits from a microsimulation modeling technique that better reproduces the natural process of Dupuytren disease through stochastic uncertainty. In addition, this modeling strategy accommodates complexity in patient-level factors, such as joint type, contracture severity, and number of fingers affected, which affect cost-effectiveness. Furthermore, numerous potential treatment regimens for recurrent Dupuytren contracture were simulated with 27 treatment permutations.

## Conclusions

In this study, LF and PNA were cost-effective treatments for managing recurrent, high-severity Dupuytren contracture of the MCP joint. For patients with recurrent single-finger, low-severity MCP joint contracture or recurrent PIP joint contractures of any severity, PNA was the only cost-effective strategy. In Dupuytren disease involving 2 low-severity MCP or PIP joint contractures, a treatment combination of PNA and LF may be cost-effective compared with repeated PNA in patients with markedly diminished quality of life. CCH was not a cost-effective treatment for Dupuytren contracture of any joint or severity.

## Notes

Supplement. **eTable 1. **One-way Sensitivity Analysis for Single-Joint Contracture From Societal Perspective of the Preferred Treatment Strategy **eTable 2. **Quality-Adjusted Life-Year and Incremental Cost-Effectiveness Ratio for Single-Joint Contracture from Health-Sector Perspective **eTable 3. **Quality-Adjusted Life-Year and Incremental Cost-Effectiveness Ratio for 2-Joint Contracture from Societal Perspective **eTable 4. **One-way Sensitivity Analysis for 2-Joint Contracture From Societal Perspective of the Preferred Treatment Strategy **eFigure 1. **Cost-effectiveness Plane **eFigure 2. **Two MCP Joint Cost-effectiveness Acceptability Curve **eFigure 3. **Two PIP Joint Cost-effectiveness Acceptability Curve
