# Supplementary material for: Cost-effectiveness of Recurrent Dupuytren Contracture Treatment
Source: JAMA Netw Open. 2020 Oct 8;3(10):e2019861. doi: 10.1001/jamanetworkopen.2020.19861 (PMC7545302; doi:10.1001/jamanetworkopen.2020.19861)
Supplement: Supplement. — eTable 1. One-way Sensitivity Analysis for Single-Joint Contracture From Societal Perspective of the Preferred Treatment Strategy eTable 2. Quality-Adjusted Life-Year and Incremental Cost-Effectiveness Ratio for Single-Joint Contracture from Health-Sector Perspective eTable 3. Quality-Adjusted Life-Year and Incremental Cost-Effectiveness Ratio for 2-Joint Contracture from Societal Perspective eTable 4. One-way Sensitivity Analysis for 2-Joint Contracture From Societal Perspective of the Preferred Treatment Strategy eFigure 1. Cost-effectiveness Plane eFigure 2. Two MCP Joint Cost-effectiveness Acceptability Curve eFigure 3. Two PIP Joint Cost-effectiveness Acceptability Curve [file jamanetwopen-e2019861-s001.pdf]

Supplemental Online Content

Yoon AP, Kane RL, Hutton DW, Chung KC. Cost-effectiveness of recurrent Dupuytren contracture treatment. *JAMA Netw Open*. 2020;3(10):e2019861. doi:10.1001/jamanetworkopen.2020.19861

eTable 1. One-way Sensitivity Analysis for Single-Joint Contracture From Societal Perspective of the Preferred Treatment Strategy
eTable 2. Quality-Adjusted Life-Year and Incremental Cost-Effectiveness Ratio for Single-Joint Contracture from Health-Sector Perspective
eTable 3. Quality-Adjusted Life-Year and Incremental Cost-Effectiveness Ratio for 2-Joint Contracture from Societal Perspective
eTable 4. One-way Sensitivity Analysis for 2-Joint Contracture From Societal Perspective of the Preferred Treatment Strategy
eFigure 1. Cost-effectiveness Plane
eFigure 2. Two MCP Joint Cost-effectiveness Acceptability Curve
eFigure 3. Two PIP Joint Cost-effectiveness Acceptability Curve

This supplemental material has been provided by the authors to give readers additional information about their work.

© 2020 Yoon AP et al. *JAMA Network Open.*

eTable 1: One-way Sensitivity Analysis for Single-Joint Contracture From Societal Perspective of the Preferred Treatment Strategy[†]

	MCP Joint Low Severity Contracture						MCP Joint High Severity Contracture						PIP Joint Low Severity Contracture						PIP Joint High Severity Contracture					
	Low			High			Low			High			Low			High			Low			High		
Willingness to Pay (1000's $)	**50**	**100**	**150**	**50**	**100**	**150**	**50**	**100**	**150**	**50**	**100**	**150**	**50**	**100**	**150**	**50**	**100**	**150**	**50**	**100**	**150**	**50**	**100**	**150**
Age	26	26	26	26	26	26	26	24	27	26	27	27	26	26	26	26	26	24	26	26	26	26	26	26
Probability of Death	26	26	26	26	26	26	26	24	27	26	24	27	26	26	26	26	26	26	26	26	26	26	26	26
**Transition Probabilities**																								
CCH Success	26	26	26	26	26	26	26	24	27	26	24	27	26	26	26	26	26	26	26	26	26	26	26	26
CCH Recurrence	26	26	26	26	26	26	26	24	27	26	24	27	26	26	26	26	26	26	26	26	26	26	26	26
PNA Success	26	26	26	26	26	26	26	27	27	26	26	27	26	26	26	26	26	26	26	26	26	26	26	26
PNA Recurrence	26	26	26	26	26	26	26	26	27	26	27	27	26	26	26	26	26	26	26	26	26	26	26	26
LF Success	26	26	26	26	26	26	26	26	27	26	27	27	26	26	26	26	26	26	26	26	26	26	26	26
LF Recurrence	26	26	26	26	26	26	26	27	27	26	26	27	26	26	26	26	26	26	26	26	26	26	26	26
**Direct Cost**																								
CCH clinic visit	26	26	26	26	26	26	26	24	27	26	24	27	26	26	26	26	26	26	26	26	26	26	26	26
CCH medication	26	26	26	26	26	26	26	24	27	26	24	27	26	26	26	26	26	26	26	26	26	26	26	26
CCH manipulation visit	26	26	26	26	26	26	26	24	27	26	24	27	26	26	26	26	26	26	26	26	26	26	26	26
CCH hand therapy	26	26	26	26	26	26	26	24	27	26	24	27	26	26	26	26	26	26	26	26	26	26	26	26
PNA procedure	26	26	26	26	26	26	26	24	27	26	24	27	26	26	26	26	26	26	26	26	26	26	26	26
PNA hand therapy	26	26	26	26	26	26	26	24	27	26	24	27	26	26	26	26	26	26	26	26	26	26	26	26
LF procedure	26	26	26	26	26	26	26	24	27	26	24	27	26	26	26	26	26	26	26	26	26	26	26	26
LF anesthesia	26	26	26	26	26	26	26	24	27	26	24	27	26	26	26	26	26	26	26	26	26	26	26	26
LF facility fee	26	26	26	26	26	26	26	27	27	26	24	27	26	26	26	26	26	26	26	26	26	26	26	26
LF hand therapy	26	26	26	26	26	26	26	24	27	26	24	27	26	26	26	26	26	26	26	26	26	26	26	26
**Indirect Cost**																								
CCH days off work	26	26	26	26	26	26	26	24	27	26	24	27	26	26	26	26	26	26	26	26	26	26	26	26
PNA days off work	26	26	26	26	26	26	26	24	27	26	27	27	26	26	26	26	26	26	26	26	26	26	26	26
LF days off work	26	26	11	26	26	26	26	27	27	26	26	27	26	26	26	26	26	26	26	26	26	26	26	26
**Complication Cost**																								
Tendon injury	26	26	26	26	26	26	26	24	27	26	24	27	26	26	26	26	26	26	26	26	26	26	26	26
Admission	26	26	26	26	26	26	26	24	27	26	24	27	26	26	26	26	26	26	26	26	26	26	26	26
Medication allergy	26	26	26	26	26	26	26	24	27	26	24	27	26	26	26	26	26	26	26	26	26	26	26	26
Skin tear	26	26	26	26	26	26	26	24	27	26	24	27	26	26	26	26	26	26	26	26	26	26	26	26
Infection	26	26	26	26	26	26	26	24	27	26	24	27	26	26	26	26	26	26	26	26	26	26	26	26

© 2020 Yoon AP et al. *JAMA Network Open.*

CRPS	26	26	26	26	26	26	26	24	27	26	24	27	26	26	26	26	26	26	26	26	26	26	26	26
Nerve injury	26	26	26	26	26	26	26	24	27	26	24	27	26	26	26	26	26	26	26	26	26	26	26	26
Artery injury	26	26	26	26	26	26	26	24	27	26	24	27	26	26	26	26	26	26	26	26	26	26	26	26
Hematoma	26	26	26	26	26	26	26	24	27	26	24	27	26	26	26	26	26	26	26	26	26	26	26	26
**Complication Rate**																								
Tendon (CCH)	26	26	26	26	26	26	26	24	27	26	24	27	26	26	26	26	26	26	26	26	26	26	26	26
Admission (CCH)	26	26	26	26	26	26	26	24	27	26	24	27	26	26	26	26	26	26	26	26	26	26	26	26
Allergy (CCH)	26	26	26	26	26	26	26	24	27	26	24	27	26	26	26	26	26	26	26	26	26	26	26	26
Skintear (CCH)	26	26	26	26	26	26	26	24	27	26	24	27	26	26	26	26	26	26	26	26	26	26	26	26
Tendon (PNA)	26	26	26	26	26	26	26	24	27	26	24	27	26	26	26	26	26	26	26	26	26	26	26	26
Infection (PNA)	26	26	26	26	26	26	26	24	27	26	24	27	26	26	26	26	26	26	26	26	26	26	26	26
CRPS (PNA)	26	26	26	26	26	26	26	24	27	26	24	27	26	26	26	26	26	26	26	26	26	26	26	26
Nerve injury (PNA)	26	26	26	26	26	26	26	24	27	26	24	27	26	26	26	26	26	26	26	26	26	26	26	26
Artery injury (PNA)	26	26	26	26	26	26	26	24	27	26	24	27	26	26	26	26	26	26	26	26	26	26	26	26
Tendon (LF)	26	26	26	26	26	26	26	24	27	26	24	27	26	26	26	26	26	26	26	26	26	26	26	26
Admission (LF)	26	26	26	26	26	26	26	24	27	26	24	27	26	26	26	26	26	26	26	26	26	26	26	26
Infection (LF)	26	26	26	26	26	26	26	24	27	26	24	27	26	26	26	26	26	26	26	26	26	26	26	26
Nerve injury (LF)	26	26	26	26	26	26	26	24	27	26	24	27	26	26	26	26	26	26	26	26	26	26	26	26
Artery injury (LF)	26	26	26	26	26	26	26	24	27	26	24	27	26	26	26	26	26	26	26	26	26	26	26	26
Hematoma (LF)	26	26	26	26	26	26	26	24	27	26	24	27	26	26	26	26	26	26	26	26	26	26	26	26
Utility (Disease state)	26	11	27	26	26	26	26	27	27	26	26	26	26	24	27	26	26	26	26	26	26	26	26	26

† Treatment Types: 11 (PNA→PNA→LF), 26 (PNA→PNA→PNA), 24 (PNA→LF→LF), 27 (LF→LF→LF)

© 2020 Yoon AP et al. *JAMA Network Open.*

eTable 2: Quality-Adjusted Life-Year and Incremental Cost-Effectiveness Ratio for Single-Joint Contracture from Health-Sector Perspective

Joint Type and Severity	Treatment Combination[1]	Average Total Cost (SDᵻ), $	Average Lifetime QALYs[2] (SDᵻ)	ICER[3] (MCSE‡),$/QALY
MCP Joint Low	PNA→PNA→PNA	2,955 (454)	15.09 (4.95)	
	PNA→PNA→LF	6,232 (1,523)	15.12 (4.97)	114,062 (11,000)
	LF→LF→LF	13,345 (2,732)	15.17 (4.98)	130,753 (18,000)
MCP Joint High	PNA→PNA→PNA	3,076 (378)	14.64 (4.80)	
	PNA→PNA→LF	6,660 (1,276)	14.72 (4.83)	43,635 (4,500)
	LF→LF→LF	13,647 (2,641)	14.88 (4.87)	45,034 (9,000)
PIP Joint Low	PNA→PNA→PNA	3,113 (329)	15.03 (4.95)	
	PNA→PNA→LF	6,788 (1,115)	15.06 (4.95)	117,877 (8,000)
	LF→LF→LF	14,114 (2,316)	15.12 (4.97)	119,422 (22,000)
PIP Joint High	PNA→PNA→PNA	3,164 (280)	14.59 (4.80)	
	PNA→PNA→LF	6,968 (956)	14.62 (4.80)	179,610 (7,000)
	LF→LF→LF	14,972 (1,741)	14.66 (4.82)	173,193 (7,500)

[1] PNA: Percutaneous Needle Aponeurotomy; LF: Limited Fasciectomy; CCH: Collagenase Clostridium Histolyticum

[2] QALY: Quality-Adjusted Life Years

[3] ICER: Incremental Cost-Effectiveness Ratio; ICER compared to next best treatment combination with highest net monetary benefit

ᵻ SD: Standard Deviation

‡ MCSE: Monte Carlo Standard Error

© 2020 Yoon AP et al. *JAMA Network Open.*

eTable 3: Quality-Adjusted Life-Year and Incremental Cost-Effectiveness Ratio for 2-Joint Contracture from Societal Perspective

Joint Type and Severity	Treatment Combination[1]	Average Total Cost (SD[ł]), $	Average Lifetime QALYs[2] (SD[ł])	ICER[3] (MCSE[‡]),$/QALY
MCP Joint Low	PNA→PNA→PNA	3,339 (544)	14.82 (4.85)	
	PNA→PNA→LF	9,700 (4,070)	14.87 (4.87)	120,999 (36,000)
	LF→LF→LF	26,235 (6,926)	14.97 (4.90)	166,081 (24,500)
MCP Joint High	PNA→PNA→PNA	3,512 (462)	13.98 (4.57)	
	PNA→PNA→LF	11,501 (3,711)	14.13 (4.61)	52,581 (6,600)
	LF→LF→LF	27,360 (7,016)	14.42 (4.70)	55,241 (13,500)
PIP Joint Low	PNA→PNA→PNA	3,567 (399)	14.59 (4.78)	
	PNA→PNA→LF	12,072 (3,267)	14.65 (4.80)	127,131 (14,500)
	LF→LF→LF	28,802 (6,583)	14.79 (4.83)	127,102 (21,667)
PIP Joint High	PNA→PNA→PNA	3,640 (338)	13.63 (4.47)	
	PNA→PNA→LF	12,867 (2,694)	13.67 (4.48)	200,653 (12,000)
	LF→LF→LF	32,386 (5,398)	13.78 (4.52)	194,195 (17,333)

[1] PNA: Percutaneous Needle Aponeurotomy; LF: Limited Fasciectomy; CCH: Collagenase Clostridium Histolyticum

[2] QALY: Quality-Adjusted Life Years

[3] ICER: Incremental Cost-Effectiveness Ratio; ICER compared to next best treatment combination with highest net monetary benefit

ł SD: Standard Deviation

‡ MCSE: Monte Carlo Standard Error

© 2020 Yoon AP et al. *JAMA Network Open.*

eTable 4: One-way Sensitivity Analysis 2-Joint Contracture From Societal Perspective of the Preferred Treatment Strategy†

	MCP Joint Low Severity Contracture						MCP Joint High Severity Contracture						PIP Joint Low Severity Contracture						PIP Joint High Severity Contracture					
	Low			High			Low			High			Low			High			Low			High		
Willingness to Pay (1000's $)	**50**	**100**	**150**	**50**	**100**	**150**	**50**	**100**	**150**	**50**	**100**	**150**	**50**	**100**	**150**	**50**	**100**	**150**	**50**	**100**	**150**	**50**	**100**	**150**
Age	26	26	11	26	11	27	26	27	27	27	27	27	26	26	27	26	27	27	26	26	26	26	22	27
Probability of Death	26	26	11	26	26	11	26	27	27	26	27	27	26	26	27	26	26	27	26	26	26	26	26	26
**Transition Probabilities**																								
CCH Success	26	26	11	26	26	11	26	27	27	26	27	27	26	26	27	26	26	27	26	26	26	26	26	26
CCH Recurrence	26	26	11	26	26	11	26	27	27	26	27	27	26	26	27	26	26	27	26	26	26	26	26	26
PNA Success	26	26	27	26	26	26	24	27	27	26	27	27	26	26	27	26	26	24	26	26	26	26	26	26
PNA Recurrence	26	26	11	26	26	27	26	27	27	24	27	27	26	26	24	26	26	27	26	26	26	26	26	26
LF Success	26	26	26	26	11	27	26	27	27	27	27	27	26	26	26	26	24	27	26	26	26	26	26	27
LF Recurrence	26	11	27	26	26	26	27	27	27	26	27	27	26	26	27	26	26	26	26	26	26	26	26	26
**Direct Cost**																								
CCH clinic visit	26	26	11	26	26	11	26	27	27	26	27	27	26	26	27	26	26	27	26	26	26	26	26	26
CCH medication	26	26	11	26	26	11	26	27	27	26	27	27	26	26	27	26	26	27	26	26	26	26	26	26
CCH manipulation visit	26	26	11	26	26	11	26	27	27	26	27	27	26	26	27	26	26	27	26	26	26	26	26	26
CCH hand therapy	26	26	11	26	26	11	26	27	27	26	27	27	26	26	27	26	26	27	26	26	26	26	26	26
PNA procedure	26	26	11	26	26	11	26	27	27	26	27	27	26	26	27	26	26	27	26	26	26	26	26	26
PNA hand therapy	26	26	11	26	26	11	26	27	27	26	27	27	26	26	27	26	26	27	26	26	26	26	26	26
LF procedure	26	26	11	26	26	11	26	27	27	26	27	27	26	26	27	26	26	27	26	26	26	26	26	26
LF anesthesia	26	26	11	26	26	11	26	27	27	26	27	27	26	26	27	26	26	27	26	26	26	26	26	26
LF facility fee	26	26	11	26	26	11	26	27	27	26	27	27	26	26	27	26	26	27	26	26	26	26	26	26
LF hand therapy	26	26	11	26	26	11	26	27	27	26	27	27	26	26	27	26	26	27	26	26	26	26	26	26
**Indirect Cost**																								
CCH days off work	26	26	11	26	26	11	26	27	27	26	27	27	26	26	27	26	26	27	26	26	26	26	26	26
PNA days off work	26	26	11	26	26	27	26	27	27	27	27	27	26	26	27	26	27	27	26	26	26	26	26	22
LF days off work	26	11	27	26	26	26	27	27	27	26	27	27	26	27	27	26	26	26	26	26	27	26	26	26
**Complication Cost**																								
Tendon injury	26	26	11	26	26	11	26	27	27	26	27	27	26	26	27	26	26	27	26	26	26	26	26	26
Admission	26	26	11	26	26	11	26	27	27	26	27	27	26	26	27	26	26	27	26	26	26	26	26	26
Medication allergy	26	26	11	26	26	11	26	27	27	26	27	27	26	26	27	26	26	27	26	26	26	26	26	26

© 2020 Yoon AP et al. *JAMA Network Open.*

Skin tear	26	26	11	26	26	11	26	27	27	26	27	27	26	26	27	26	26	27	26	26	26	26	26	26
Infection	26	26	11	26	26	11	26	27	27	26	27	27	26	26	27	26	26	27	26	26	26	26	26	26
CRPS	26	26	11	26	26	11	26	27	27	26	27	27	26	26	27	26	26	27	26	26	26	26	26	26
Nerve injury	26	26	11	26	26	11	26	27	27	26	27	27	26	26	27	26	26	27	26	26	26	26	26	26
Artery injury	26	26	11	26	26	11	26	27	27	26	27	27	26	26	27	26	26	27	26	26	26	26	26	26
Hematoma	26	26	11	26	26	11	26	27	27	26	27	27	26	26	27	26	26	27	26	26	26	26	26	26
**Complication Rate**																								
Tendon (CCH)	26	26	11	26	26	11	26	27	27	26	27	27	26	26	27	26	26	27	26	26	26	26	26	26
Admission (CCH)	26	26	11	26	26	11	26	27	27	26	27	27	26	26	27	26	26	27	26	26	26	26	26	26
Allergy (CCH)	26	26	11	26	26	11	26	27	27	26	27	27	26	26	27	26	26	27	26	26	26	26	26	26
Skin tear (CCH)	26	26	11	26	26	11	26	27	27	26	27	27	26	26	27	26	26	27	26	26	26	26	26	26
Tendon (PNA)	26	26	11	26	26	11	26	27	27	26	27	27	26	26	27	26	26	27	26	26	26	26	26	26
Infection (PNA)	26	26	11	26	26	11	26	27	27	26	27	27	26	26	27	26	26	27	26	26	26	26	26	26
CRPS (PNA)	26	26	11	26	26	11	26	27	27	26	27	27	26	26	27	26	26	27	26	26	26	26	26	26
Nerve injury (PNA)	26	26	11	26	26	11	26	27	27	26	27	27	26	26	27	26	26	27	26	26	26	26	26	26
Artery injury (PNA)	26	26	11	26	26	11	26	27	27	26	27	27	26	26	27	26	26	27	26	26	26	26	26	26
Tendon (LF)	26	26	11	26	26	11	26	27	27	26	27	27	26	26	27	26	26	27	26	26	26	26	26	26
Admission (LF)	26	26	11	26	26	11	26	27	27	26	27	27	26	26	27	26	26	27	26	26	26	26	26	26
Infection (LF)	26	26	11	26	26	11	26	27	27	26	27	27	26	26	27	26	26	27	26	26	26	26	26	26
Nerve injury (LF)	26	26	11	26	26	11	26	27	27	26	27	27	26	26	27	26	26	27	26	26	26	26	26	26
Artery injury (LF)	26	26	11	26	26	11	26	27	27	26	27	27	26	26	27	26	26	27	26	26	26	26	26	26
Hematoma (LF)	26	26	11	26	26	11	26	27	27	26	27	27	26	26	27	26	26	27	26	26	26	26	26	26
Utility (Disease state)	26	27	27	26	26	26	27	27	27	26	27	27	26	27	27	26	26	26	26	26	27	26	26	26

† Treatment Types: 11 (PNA→PNA→LF), 22 (LF→PNA→PNA), 26 (PNA→PNA→PNA), 24 (PNA→LF→LF), 27 (LF→LF→LF)

© 2020 Yoon AP et al. *JAMA Network Open.*

eFigure 1. Cost-effectiveness Plane

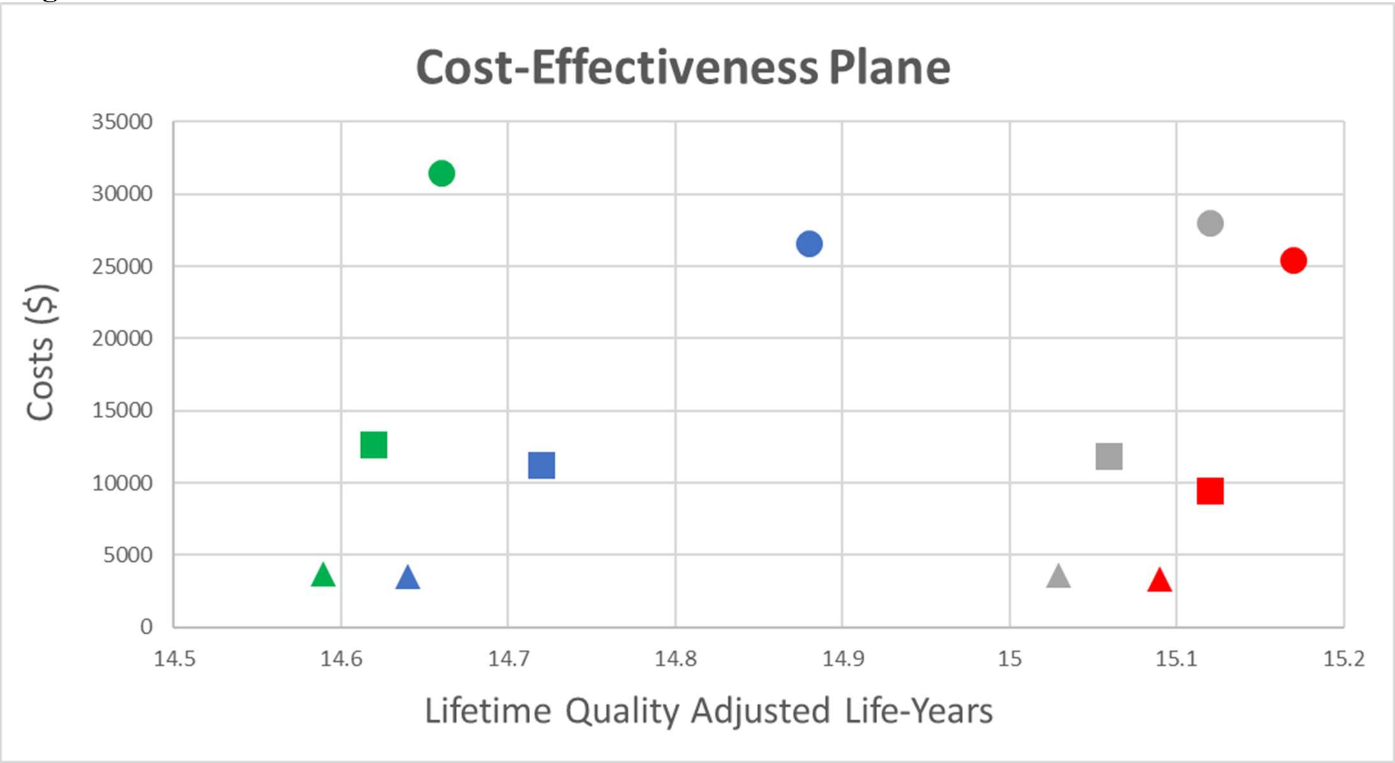

Triangle: PNA→PNA→PNA; Square: PNA→PNA→LF; Circle: LF→LF→LF
Red: MCP joint low severity contracture; Blue: MCP joint high severity contracture; Gray: PIP joint low severity contracture; Green: PIP joint high severity contracture

© 2020 Yoon AP et al. *JAMA Network Open.*

eFigure 2. Two MCP Joint Cost-effectiveness Acceptability Curve

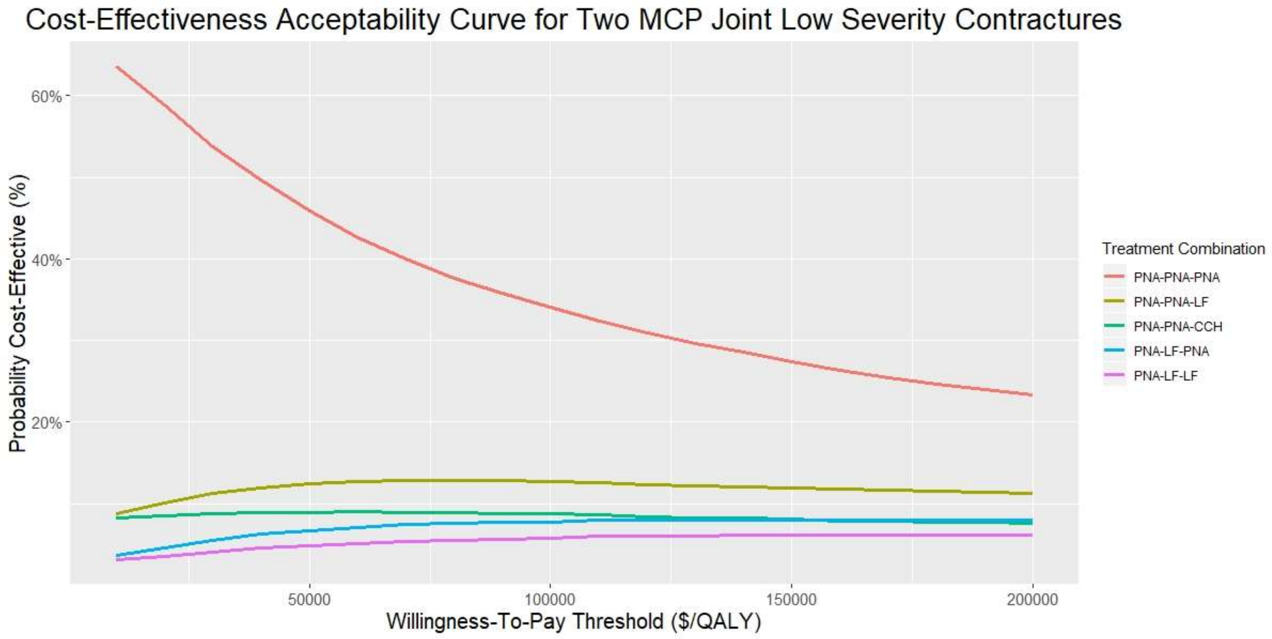

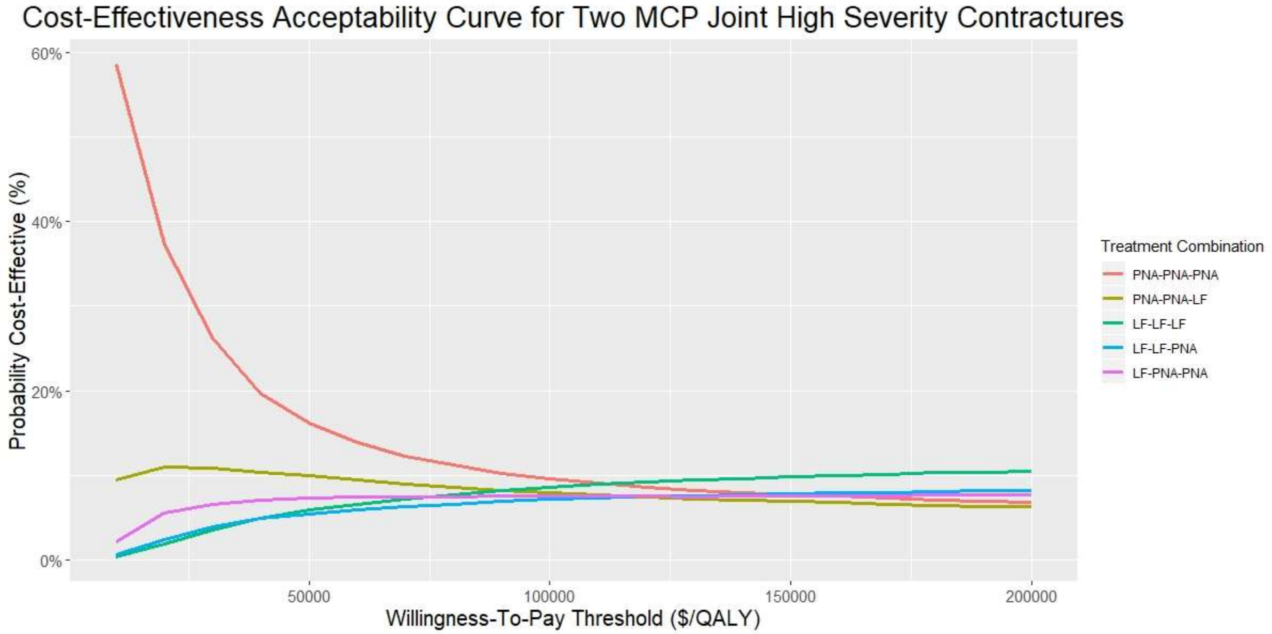

© 2020 Yoon AP et al. *JAMA Network Open.*

eFigure 3. Two PIP Joint Cost-effectiveness Acceptability Curve

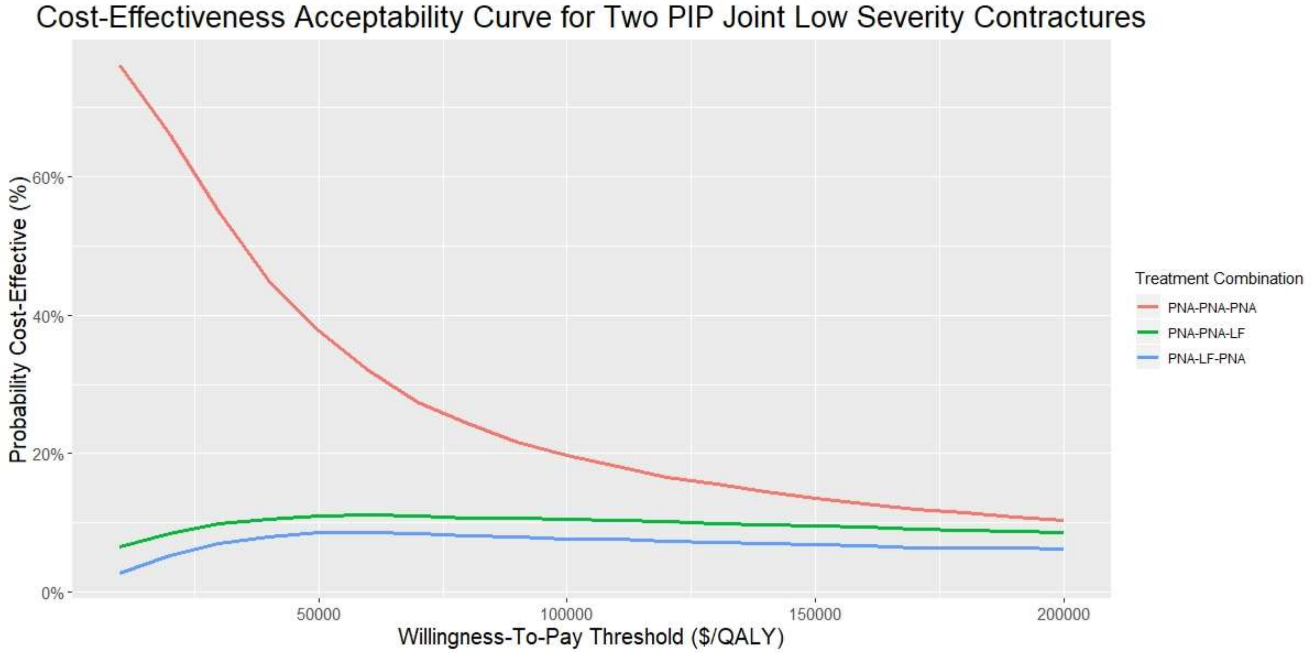

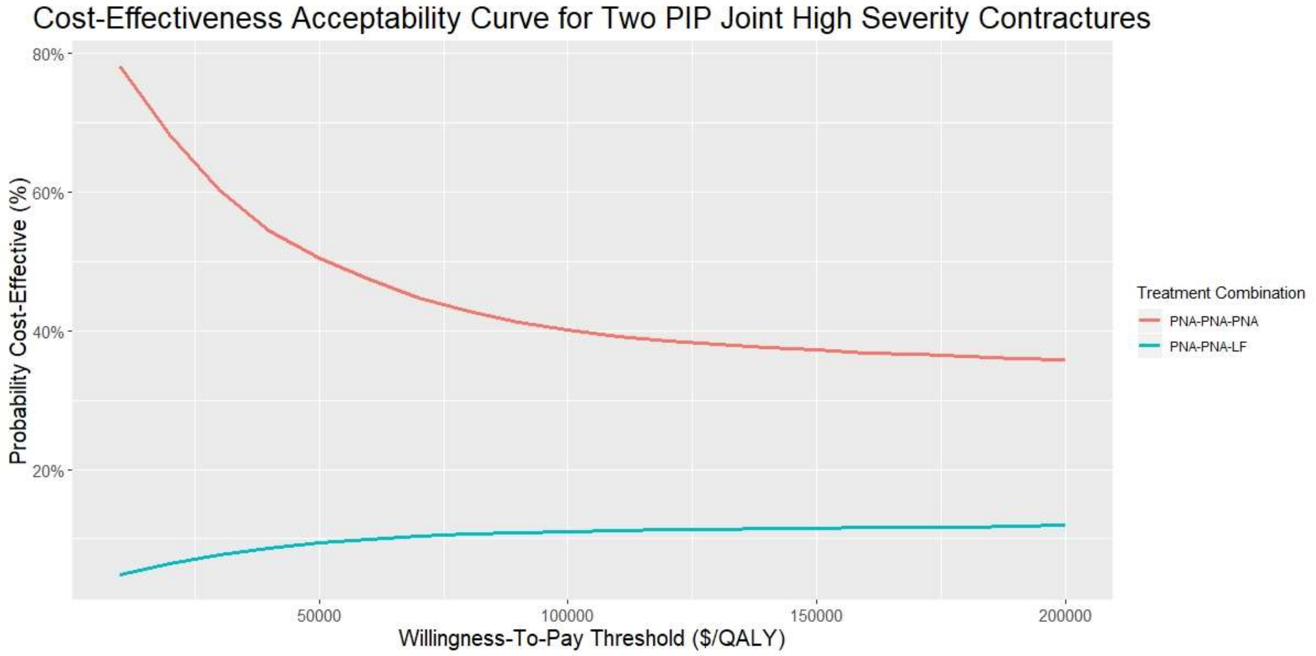

© 2020 Yoon AP et al. *JAMA Network Open.*
